# Noninvasive Quantification of Glucose Metabolism in Mice Myocardium Using the Spline Reconstruction Technique

**DOI:** 10.3390/jimaging10070170

**Published:** 2024-07-16

**Authors:** Alexandros Vrachliotis, Anastasios Gaitanis, Nicholas E. Protonotarios, George A. Kastis, Lena Costaridou

**Affiliations:** 1 Department of Medical Physics, School of Medicine, University of Patras, 26504 Patras, Greece; vr.alexander@hotmail.com (A.V.); costarid@upatras.gr (L.C.); 2 Clinical, Experimental Surgery & Translational Research, Biomedical Research Foundation (BRFAA), Academy of Athens, 4 Soranou Ephessiou, 11527 Athens, Greece; agaitanis@bioacademy.gr; 3 Mathematics Research Center, Academy of Athens, 11527 Athens, Greece; nprotonotarios@academyofathens.gr; 4 Institute of Nuclear & Radiological Sciences & Technology, Energy & Safety, National Center for Scientific Research “Demokritos”, 15341 Athens, Greece

**Keywords:** dynamic small-animal imaging, image reconstruction, PET, Mediso nanoScan® PC (PET8/2) PET/CT, myocardial glucose uptake quantification, SRT

## Abstract

The spline reconstruction technique (SRT) is a fast algorithm based on a novel numerical implementation of an analytic representation of the inverse Radon transform. The purpose of this study was to compare the SRT, filtered back-projection (FBP), and the Tera-Tomo 3D algorithm for various iteration numbers, using small-animal dynamic PET data obtained from a Mediso nanoScan^®^ PET/CT scanner. For this purpose, Patlak graphical kinetic analysis was employed to noninvasively quantify the myocardial metabolic rate of glucose (MRGlu) in seven male C57BL/6 mice (n=7). All analytic reconstructions were performed via software for tomographic image reconstruction. The analysis of all PET-reconstructed images was conducted with PMOD software (version 3.506, PMOD Technologies LLC, Fällanden, Switzerland) using the inferior vena cava as the image-derived input function. Statistical significance was determined by employing the one-way analysis of variance test. The results revealed that the differences between the values of MRGlu obtained via SRT versus FBP, and the variants of he Tera-Tomo 3D algorithm were not statistically significant (*p* > 0.05). Overall, the SRT appears to perform similarly to the other algorithms investigated, providing a valid alternative analytic method for preclinical dynamic PET studies.

## 1. Introduction

Positron emission tomography (PET) is a quantitative, molecular imaging modality with a vast range of clinical applications in oncology [1], neurology [2], cardiology [3], as well as psychiatry [4]. To improve diagnosis and treatment monitoring, PET is combined with computed tomography (CT) or magnetic resonance imaging (MRI) to provide both functional and anatomical information. Lately, PET/CT imaging has also contributed to COVID-19 research [5]. Furthermore, small-animal PET/CT is a well-established imaging modality in preclinical research [6] as well as in drug development and discovery [7].

**Figure 1:**
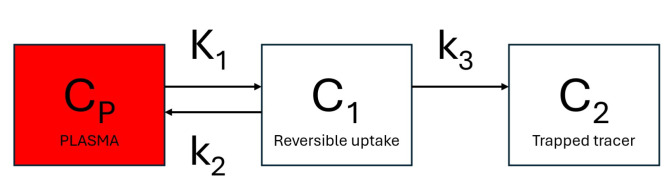
Schematic presentation of a 3-compartment model with one input function. $C_{p}$ is the tracer concentration in arterial blood. Compartment $C_{1}$ represents the free and nondisplaceable part of the tracer into the tissue, and compartment $C_{2}$ represents the specific bound part of the tracer.

Dynamic PET can provide reliable assessments of dedicated metabolic steps of metabolic active radiotracers, such as the glucose analog ^18^F-fluorodeoxyglucose (^18^F-FDG) [8]. ^18^F-FDG enters the cell via glucose transporters ($K_{1}$ and $k_{2}$ rate constants) and is phosphorylated by hexokinase to ^18^F-FDG-6-phosphate ($k_{3}$ rate constant). The phosphorylation prevents the glucose from being released again from the cell, trapping the radiolabeled product within it (Figure 1). The ^18^F-FDG uptake rate constant, $K_{i}=\left(K_{1}\times k_{3}\right)/\left(k_{2}+k_{3}\right)$, can be estimated using Patlak graphical kinetic analysis, assuming there is negligible tracer washout ($k_{4}=0$) [9]. ^18^F-FDG has been modeled extensively with Patlak analysis in humans [10], rats [11,12], and mice [13,14].

The quantification of the metabolic rate of glucose (MRGlu) from ^18^F-FDG PET requires the knowledge of the arterial input function (AIF) to measure the radio-ligand concentration in plasma as a function of time. The gold standard for the determination of the AIF is repeated arterial blood sampling [14,15]. However, such an approach in mice is technically challenging as it is difficult to insert a cannula into a small artery and then draw multiple blood samples without affecting their physiology. Moreover, the limited blood volume (∼2 mL) in small-sized animals makes it practically impossible to perform accurate blood sampling [14]. A noninvasive approach for overcoming these difficulties is based on the extraction of the input function from dynamic PET images [12,14,15,16]. The high spatial resolution of modern small-animal PET scanners allows the acquisition of image-derived input functions (IDIFs) even from small anatomical regions. Several studies have suggested that the inferior vena cava (IVC) provides a reliable and reproducible IDIF for the Patlak analysis of myocardial glucose uptake in mice [13,14].

Image reconstruction plays a vital role in the quantification of MRGlu from ^18^F-FDG PET experiments, especially when an IDIF is employed. The traditional reconstruction algorithms include (a) the filtered back-projection (FBP) algorithm [17] and (b) iterative reconstruction methods such as maximum likelihood expectation maximization (MLEM) [18] and its accelerated successor, ordered subset expectation maximization (OSEM) [19]. FBP offers a direct mathematical solution for the formation of an image but suffers from high noise and streak artifacts. Iterative methods use the system model and regularizations to reconstruct images with superior image quality, contrast, signal-to-noise ratio (SNR), and resolution compared to FBP [20]. However, despite their advantages, these methods have several ambiguities. Firstly, iterative methods have certain limitations regarding image quantification due to the substantial positive bias [21]. Secondly, the accuracy of the system model affects the reconstructed images. An accurate system model requires point sources to measure the point spread function (PSF) of the scanner, which is time consuming and expensive [21]. Lastly, there exist several ambiguities such as stopping the algorithms at the proper number of iterations [22,23] as well as selecting the best regularization [21]. It is important to note that 3D-OSEM has now become the standard reconstruction method for routine static and dynamic PET imaging, widely used by most commercial clinical and preclinical systems. However, FBP still has clinical and preclinical value, especially for quantitative, dynamic PET brain studies [24].

The spline reconstruction technique (SRT) is an image reconstruction algorithm based on an analytic formulation for the inverse Radon transform [25,26]. It involves the numerical computation of an integral of the Hilbert transform of a sinogram through an approximation using custom-made cubic splines. Formulated in the physical space, the SRT allows restricting the reconstruction only within the object pixels by employing mathematical symmetries to eliminate streak artifacts outside the object boundary (sinogram thresholding). This optional feature can also be used to reduce reconstruction time. The SRT can be applied to various imaging techniques such as PET, CT, and SPECT [27]. Its detailed mathematical derivation is presented elsewhere [28]. In previous PET studies, the SRT was evaluated by using simulated data for a clinical PET system and real data obtained from clinical and preclinical PET scanners via static acquisition [28,29,30]. According to these studies, the SRT appears to be promising for the evaluation of myocardial viability. Furthermore, at the expense of slightly increased noise in the reconstructed images, the SRT could be useful for the quantification of small hot regions of interest (ROIs), cold ROIs, as well as in low-count imaging applications.

The purpose of this study was to compare SRT, FBP, and Tera-Tomo 3D (a version of OSEM algorithm) for various iteration numbers, using preclinical dynamic PET data. More specifically, the performance of the algorithms was evaluated based on the quantification of the slope $K_{i}$ and MRGlu in the mouse myocardium.

## 2. Materials and Methods

### 2.1. Imaging System

The imaging system used in this study was a Mediso nanoScan^®^ PC (PET8/2) PET/CT scanner (Mediso Medical Imaging Systems, Budapest, Hungary), comprising two rings with inner diameter of 12.6 cm. Each ring had eight detector modules, with each module consisting of 29 × 29 LYSO crystal needles with dimensions 1.51 mm^3^ × 1.51 mm^3^ × 10 mm^3^. The scanner’s axial and transaxial fields of view were 98.6 mm and 80 mm, respectively. Detailed technical characteristics of the imaging system are presented in [31].

### 2.2. Animal Model

Seven healthy, 3-month-old, male C57BL/6 mice (n=7), with an average weight of 26.12 ± 1.78 g, were imaged via the aforementioned imaging system. The animals were food-deprived for eight hours (8 h) prior to imaging but were allowed water ad libitum. All mice were housed in the Laboratory Animal Facility of BRFAA, in compliance with the National and European Legislation for the protection of animals used for experimental purposes as well as the guidelines of the Association for the Assessment and Accreditation of Laboratory Animal Care (AAALAC).

### 2.3. PET Imaging

The levels of blood glucose were measured before and after imaging by taking a small blood sample from the tail vein. According to Equation (8), these blood glucose levels are necessary for the calculation of the metabolic MRGlu values. The animals were anesthetized under standard isoflurane anesthesia, 2% isoflurane in ∼1 L/min oxygen, and remained immobilized on the scanning bed via an adjusted mask positioned on their face. A constant body temperature (∼36 °C) was maintained half an hour before as well as during the experiment. An average of 8.09 ± 0.99 MBq of ^18^F-FDG was administered via the tail vein. Dynamic 60 min PET scans were performed with a combination of 5 min whole-body CT scans (X-ray: 50 kVP). Breathing rate and body temperature were used to continuously monitor the animals throughout the PET/CT scans.

### 2.4. PET Data Acquisition

List-mode PET data were acquired right after tracer administration and framed into a dynamic sequence of 5 × 2, 4 × 5, 3 × 10, 8 × 30, 5 × 60, 4 × 300, and 3 × 600 s frames [14]. The Fourier rebinning algorithm (FORE) was applied to the list-mode PET data to generate two-dimensional (2D) sinograms [32], which are necessary for the analytic reconstructions. The maximal FORE ring difference was set to 16, and the energy window was set to 400–600 keV. The oversampling rate (blur factor) of the line-of-response (LOR) endpoints in the physical volume during rebinning was set to the default value of 30. The acquired sinograms were corrected for attenuation, scatter, randoms, and decay reference time. The dimensions of each sinogram sdfd 284 detectors × 512 angles, with 123 image slices and a bin size of 0.3 mm.

### 2.5. Reconstructions

#### 2.5.1. Analytic Reconstruction Methods

All rebinned (2D) sinograms were reconstructed using the FBP and SRT algorithms. These reconstructions were performed with the Software for Tomographic Image Reconstruction (STIR, version 3.0) open-source library [33]. The reconstruction grid for the images generated was 285 × 285 × 123, with scale factors of 0.3 mm/pixel × 0.3 mm/pixel × 0.795 mm/pixel, respectively. No filtering or smoothing was applied to the reconstructed images afterreconstruction.

The FBP reconstruction algorithm is well known. The inverse Radon transform implemented via the FBP algorithm is expressed by the following formula:

(1) $$f\left(x_{1},x_{2}\right)=\frac{1}{N}\sum_{n=0}^{N-1}s^{*}\left(\rho ,\theta_{n}\right),$$

where

(2) $$s^{*}\left(\rho ,\theta \right)=\frac{1}{2\pi }F^{-1}\left\{S\left(\xi_{\rho },\theta \right)*H\left(\xi_{\rho }\right)\right\},$$

F and $F^{-1}$ denote the direct and inverse Fourier transform, $S(\xi_{\rho };,\theta )$ is the sinogram in the spatial frequency domain given by the expression:

(3) $$S\left(\xi_{\rho },\theta \right)=F\left\{\hat{f}\left(\rho ,\theta \right)\right\},$$

and the function $H(\xi_{\rho })$ denotes some appropriate filter function. For our comparisons, $H(\xi_{\rho })$ is a ramp filter with a cutoff frequency equal to the Nyquist frequency.

The SRT algorithm assumes a simple geometrical model of parallel rays similar to FBP. It is based on the application of cubic interpolating splines for the calculation of the inverse Radon transform. The inverse Radon transform using the SRT of a function $\hat{f}\left(\rho ,\theta \right)$ (sinogram) is given by the expression

(4) $$\begin{array}{c} f\left(x_{1},x_{2}\right)=-\frac{1}{4\pi^{2}}\int_{0}^{2\pi }\left\{C\left(\theta \right)+\frac{1}{2}\left({\hat{f}}_{n}^{\prime \prime }-{\hat{f}}_{1}^{\prime \prime }\right)\rho +D_{n-1}\left(\rho ,\theta \right)\ln|\rho -\rho_{n}|-D_{1}\left(\rho ,\theta \right)\ln\left|\rho -\rho_{1}\right|\right.\\ \left.+\sum_{i=1}^{n-2}\left[D_{i}\left(\rho ,\theta \right)-D_{i+1}\left(\rho ,\theta \right)\right]\ln\left|\rho -\rho_{i+1}\right|\right\}d\theta , \end{array}$$

where ρ is the distance from the origin, and θ is the angle of rotation. The term ${\hat{f}}_{n}^{\prime \prime }$ represents the second derivative with respect to ρ of $\hat{f}\left(\rho ,\theta \right)$, evaluated at $\rho_{n}$. The terms C(θ) and $D_{i}\left(\rho ,\theta \right)$, given by Equations (15a) and (15b) in [28], can be expressed as functions of ${\hat{f}}_{i}$ and ${\hat{f}}_{i}^{\prime \prime }$. Furthermore, we note that C(θ) is a term independent of ρ, and $D_{i}\left(\rho ,\theta \right)$ is the first derivative of the spline approximation of $\hat{f}\left(\rho ,\theta \right)$ with respect to ρ in the interval $\left[\rho_{i},\rho_{i+1}\right]$. The second derivatives with respect to ρ of $\hat{f}\left(\rho ,\theta \right)$, denoted by ${\hat{f}}_{i}^{\prime \prime }$ and evaluated at $\rho_{i}$, can be obtained directly from ${\hat{f}}_{i}$. We note that for the computation of the first and second derivatives of $\hat{f}\left(\rho ,\theta \right)$, we follow the procedure of [28]; namely, we solve the system of equations that arises from the continuity of the explicitly given first and second derivatives of the local interpolating splines, respectively. The integral over θ corresponds to a back-projection operation. The sinogram thresholding feature was not employed for the reconstructions performed via the SRT.

#### 2.5.2. Iterative Reconstruction Method

The list-mode PET data were reconstructed using the Tera-Tomo 3D image reconstruction algorithm, which is the commercial reconstruction algorithm provided with the Mediso nanoScan^®^ PC (PET8/2) PET/CT scanner. The data were corrected for scatter, randoms, dead time, decay, attenuation, axial sensitivity, and normalization. The reconstruction grid for the images generated was 283 × 283 × 328, with scale factors of 0.3 mm/pixel × 0.3 mm/pixel × 0.3 mm/pixel, respectively. No filtering or smoothing was applied to the reconstructed images after reconstruction.

The Tera-Tomo 3D algorithm is an iterative image reconstruction method that involves the OSEM algorithm [34]. Its mathematical formulation is given by the expression

(5) $$x_{i}^{\left(n+1\right)}=\frac{x_{i}^{\left(n\right)}}{\sum_{j\in S_{k}}a_{ij}+\beta \frac{\partial TV(x^{\left(n\right)}\left(u\right))}{\partial x}}\sum_{j\in S_{k}}\frac{a_{ij}y_{j}}{\sum_{i=1}^{I}a_{ij}x_{j}^{\left(n\right)}},$$

where

(6) $$TV\left(x\right)=\int \sqrt{{\left|\nabla x\left(u\right)\right|}^{2}}du,$$

$a_{ij}$ is the system matrix, $S_{k}$ is the subset number, and $y_{j}$ represents the measured LOR counts. The term β is kept relatively small to maintain the non-negativity property of the algorithm. When β=0, the algorithm is converted to the usual OSEM algorithm. The purpose of the total variation (TV) term is edge preservation. Minimizing this term smooths the image without introducing the Gibbs phenomenon. The reconstruction parameters used for the Tera-Tomo 3D algorithm were 4 subsets-5 iterations (Tera-Tomo 4-5) and 4 subsets-13 iterations (Tera-Tomo 4-13). The level of regularization was set to high (HR-$\beta =10^{-3}$). As indicated in [31], a high regularization level at 52 iteration updates (4 subsets-13 iterations) and 30 min acquisition duration were found to optimize its performance.

### 2.6. Image Analysis

**Figure 2:**
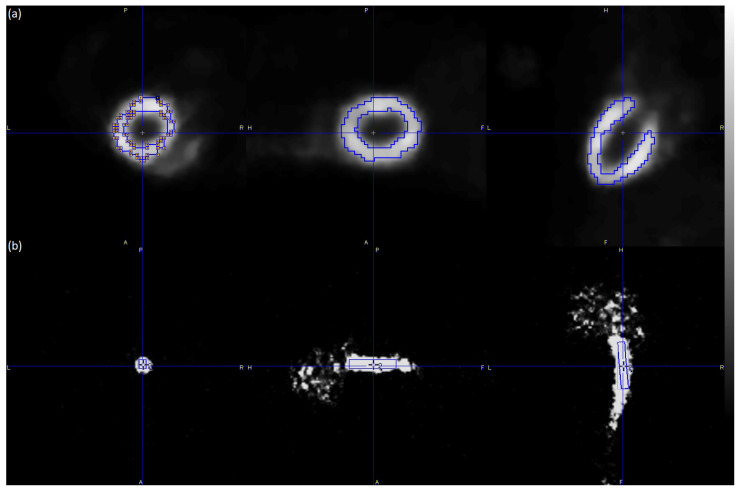
Illustration of the ROIs and VOI defined for the hot region of the myocardium (**a**) and the hot value voxels of the IVC (**b**) in a representative male C57BL/6 mouse. The myocardium ROIs were selected using late frames (frames 27–32) of the PET scan, while the IVC VOI was selected using short-duration (2 s) early frames (frames 1–3) of the PET scan. The figure was created via PMOD software [35]. The scalar values in the color bar range from zero (black) to the maximum (white) of the reconstruction algorithm Tera-Tomo 4–13. A = anterior, F = feet, H = head, L = left, P = posterior, and R = right.

The Patlak graphical kinetic analysis of the PET reconstructed images (a total of 202,048 images) was performed via PMOD computer software (version 3.506, PMOD Technologies LLC, Fällanden, Switzerland) [35], which is known as the reference tool for PET kinetic modeling. For each reconstruction algorithm, manual ROIs were defined for the entire hot region of the myocardium (tissue). Additionally, a cube volume of interest (VOI) was defined for the hot value voxels of the IVC (whole blood) as shown in Figure 2.

The Patlak plot [36], also known as Patlak–Gjedde plot, belongs to a group of graphical analysis techniques and its mathematical formulation is given by the expression:

(7) $$\frac{C_{T}\left(t\right)}{C_{P}\left(t\right)}=K\frac{\int_{0}^{t}C_{P}\left(\tau \right)d\tau }{C_{P}\left(t\right)}+V,$$

with $C_{P}\left(t\right)$ representing the input curve, $C_{T}\left(t\right)$ the measured tissue time–activity curve (TAC), *K* the slope, and *V* the intercept. The interpretation of *K* and *V* is based on the underlying compartment model. For the ^18^F-FDG model, *K* equals $K_{i}=\left(K_{1}\times k_{3}\right)/\left(k_{2}+k_{3}\right)$, while $V=V_{0}+vB$, where $V_{0}$ represents the distribution volume of the reversible compartment $C_{1}$ and vB the fractional blood volume. For systems with irreversible compartments, this plot results in a straight line after an equilibration time ($t^{*}$). The option “fit all regions” was activated in PMOD computer software to apply the analysis to the same data segment in all regions. Both slope $K_{i}$ (mL/ccm/min) and MRGlu (μmol/min/100 g) were calculated automatically using PMOD computer software. MRGlu is subsequently obtained from slope $K_{i}$ [8] according to the expression:

(8) $$MRGlu=K_{i}\frac{BG}{LC},$$

where BG is the average blood glucose concentration measured at the start and at the end of the scan (mmol/L), and LC is the lumped constant, equaling 0.67 as estimated for rodents [14].

### 2.7. Statistical Analysis

Statistical significance was determined by employing the one-way analysis of variance (ANOVA) test, based on the accepted normality of the data distribution. The ANOVA tests the null hypothesis, which stated that samples in all groups are drawn from populations with the same mean values. Its nonparametric equivalent is the Kruskal–Wallis test. For the comparisons between FBP, SRT, and the variants of Tera-Tomo 3D, a *p* value of less than 0.05 was considered statistically significant. Statistical analyses were completed using MedCalc software (version 18.9.1, MedCalc Software Ltd., Ostend, Belgium) [37].

## 3. Results

**Figure 3:**
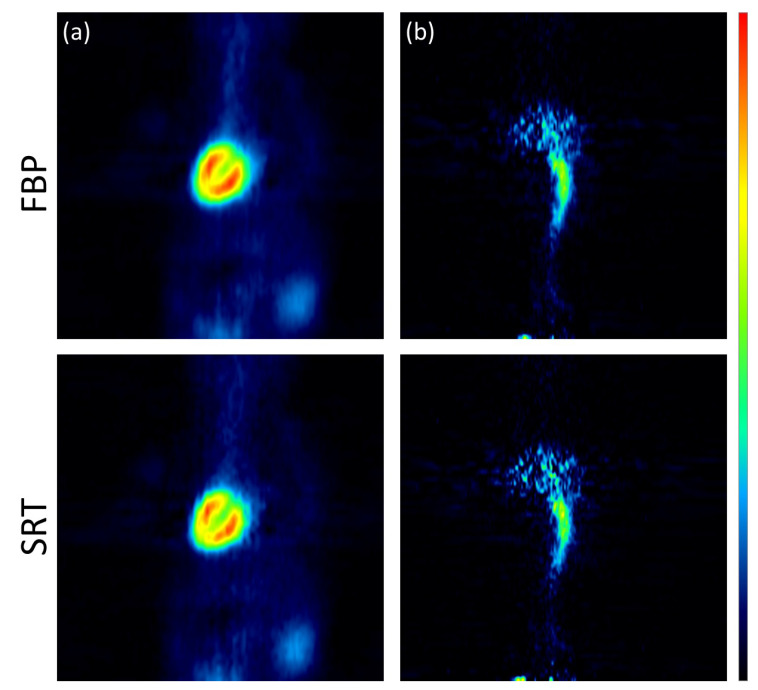
Analytic reconstructions using FBP and SRT. Coronal view of the myocardium (**a**) and the IVC (**b**) in a representative male C57BL/6 mouse. The figure was created via PMOD software [35]. The scalar values in the color bar range from zero (black) to the maximum (red) of each reconstruction algorithm.

**Figure 4:**
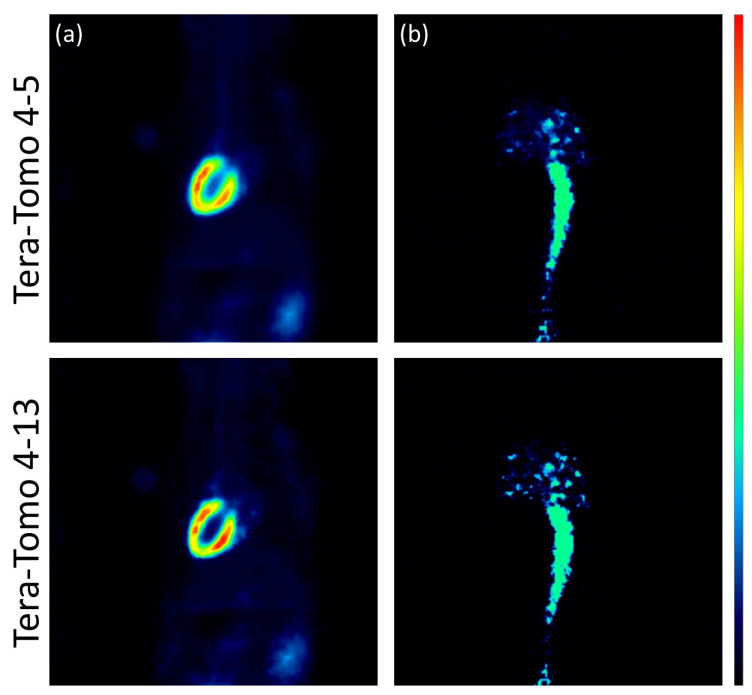
Iterative reconstructions using the two variants of the Tera-Tomo 3D algorithm, namely, Tera-Tomo 4-5 and Tera-Tomo 4-13. Coronal view of the myocardium (**a**) and the IVC (**b**) in a representative male C57BL/6 mouse. The figure was created via PMOD software [35]. The scalar values in the color bar range from zero (black) to the maximum (red) of each reconstruction algorithm.

The images reconstructed using the analytic algorithms FBP and SRT are presented in Figure 3. The images reconstructed using the two variants of the Tera-Tomo 3D algorithm are presented in Figure 4. There were no visual differences between the reconstructed images obtained for the seven animals; therefore, images from a representative animal are presented. Subjective visual inspection identifies small differences in noise texture between FBP, SRT, and the Tera-Tomo 3D reconstructions. As expected, both FBP and SRT produced slightly noisier images compared to those produced by the variants of the Tera-Tomo 3D algorithm. These observations are in agreement with the ones presented in [30].

Table 1 presents the measured metabolic value slope $K_{i}$ (mL/ccm/min), and Table 2 provides the measured metabolic value MRGlu (μmol/min/100 g) of the applied Patlak kinetic model for all animals and for the four reconstruction algorithms employed, namely, FBP, SRT, Tera-Tomo 4-5, as well as Tera-Tomo 4-13.

**Figure 5:**
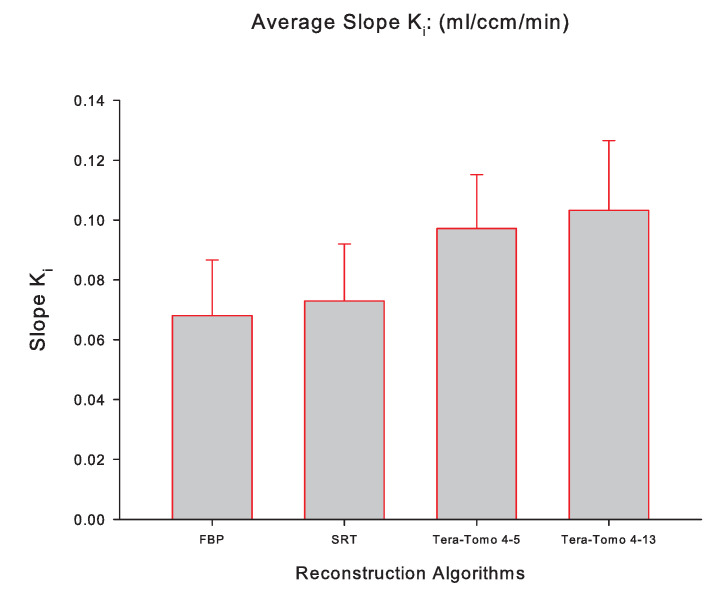
The averaged slope $K_{i}$ (mL/ccm/min) of the applied Patlak kinetic model for all four reconstruction algorithms employed, namely, FBP, SRT, Tera-Tomo 4-5, and Tera-Tomo 4-13. The averaged slope $K_{i}$ was calculated via PMOD software [35]. The results are illustrated as mean ± SD.

**Figure 6:**
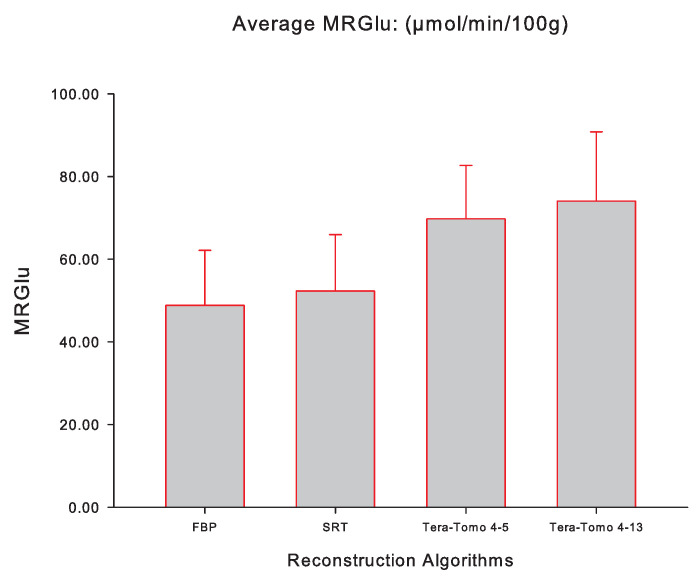
The averaged MRGlu (μmol/min/100 g) of the applied Patlak kinetic model for all four reconstruction algorithms employed, namely, FBP, SRT, Tera-Tomo 4-5, and Tera-Tomo 4-13. The averaged MRGlu was calculated via PMOD software [35]. The results are illustrated as mean ± SD.

Figure 5 illustrates the averaged slope $K_{i}$ (mL/ccm/min), while Figure 6 illustrates the averaged MRGlu (μmol/min/100 g) for FBP, SRT, Tera-Tomo 4-5, and Tera-Tomo 4-13 measured via PMOD computer software in the myocardium of each animal model (seven male C57BL/6 mice). The results are presented as the mean ± SD. The ANOVA test was employed to determine statistical significance (Shapiro–Wilk test for normal distribution: p=0.2845).

Table 1 and Table 2 and Figure 5 and Figure 6 indicate that the images obtained via SRT exhibit numerically higher slope $K_{i}$ and MRGlu values compared to the images obtained via FBP. Furthermore, the reconstructions obtained via the variants of the Tera-Tomo 3D algorithm exhibit notably higher slope $K_{i}$ and MRGlu values compared to the ones obtained via FBP and SRT algorithms (Figure 5 and Figure 6). Additionally, in the Tera-Tomo 3D reconstructions, an increase in both metabolic values occurred as the number of iterations increased (Figure 5 and Figure 6). The ANOVA test indicated that the differences observed between the values of slope $K_{i}$ and MRGlu obtained via SRT versus FBP, Tera-Tomo 4-5, and Tera-Tomo 4-13 reconstructions were not statistically significant (*p* > 0.05). This was also true for the FBP and Tera-Tomo 4-5 reconstructions (*p* > 0.05). However, it is important to note that statistically significant differences (p=0.011) were observed for both metabolic values when comparing the FBP and Tera-Tomo 4-13 reconstructions. Lastly, the images obtained via the two variatnts of the Tera-Tomo 3D algorithm, namely, Tera-Tomo 4-5 and Tera-Tomo 4-13, exhibited no statistically significant differences (*p* > 0.05) regarding the estimation of slope $K_{i}$ and MRGlu metabolic values.

## 4. Discussion

In this work, the SRT, an analytic image reconstruction algorithm, was evaluated for the quantification of the glucose metabolism in the mouse myocardium. For this purpose, Patlak graphical kinetic analysis was employed to noninvasively quantify the slope $K_{i}$ and MRGlu of the mouse myocardium. The SRT was compared to FBP and Tera-Tomo 3D for various iteration numbers, using small-animal dynamic PET data acquired with a preclinical PET/CT scanner. It is essential to note that the reconstructed myocardial images incorporated a blurring component due to the noncorrection of cardiac motion.

The results indicated that the reconstructions obtained via the SRT and FBP algorithms exhibited no statistically significant differences between the slope $K_{i}$ and MRGlu values. More specifically, the SRT images exhibited numerically higher metabolic values than those produced by the FBP analytic method. This is probably due to the fact that the SRT operates exclusively in the image (physical) space, without incorporating the Fourier transform, which is expected to benefit the recovery coefficient (RC) of the reconstructed images. RC quantifies the “activity recovery” in environments with zero activity background so is therefore indicative of the spatial resolution of the imaging system [38,39]. As presented in [30], SRT reconstructions exhibit higher RC values than those of FBP at various acquisition durations, namely, 12, 60, 300, 600, and 1200 s. It should be mentioned that the use of short-duration time frames is particularly important when trying to derive an IDIF. In this study, IDIFs were calculated using the first three 2 s frames (0–2 s, 2–4 s, and 4–6 s) of the IVC. The shorter early frames used may allow sharper definition of the IVC activity peak and clearance [14]. Lastly, it should be noted that the IVC provides a reliable and reproducible IDIF for the Patlak analysis of myocardial glucose uptake in mice [14].

The analysis performed via MedCalc computer software revealed that the reconstructions obtained via the SRT and the variants of the Tera-Tomo 3D algorithm exhibited no statistically significant differences in the estimation of the slope $K_{i}$ and MRGlu values. More specifically, Tera-Tomo 4-5 images and Tera-Tomo 4-13 images exhibited numerically higher slope $K_{i}$ and MRGlu values compared to those produced by the SRT. Furthermore, in the Tera-Tomo 3D reconstructions, an increase in both metabolic values occurred as the number of iterations increased. Lastly, no major visual differences were observed between the reconstructed images obtained via the two variants of the Tera-Tomo 3D algorithm (Figure 4).

It is essential to mention that the differences observed in the values of both Patlak parameters obtained via FBP and Tera-Tomo 4-5 were not statistically significant. However, this was not true for the corresponding differences observed between FBP and Tera-Tomo 4-13. A possible explanation for this could be that MLEM and its variant, OSEM, have been shown to produce bias (positivity constraint) in applications where images are reconstructed from a relatively small number of counts [28]. This bias might affect the quantification accuracy of dynamic PET imaging using short-duration time frames (i.e., IVC IDIF). Yet, this is not expected to significantly affect the imaging of regions with moderate activity at later, longer time frames, such as the myocardium after $t^{*}$. Furthermore, FBP and the SRT exhibit a negative bias that might also affect the quantification accuracy in dynamic PET imaging. However, as presented in [28], the SRT provides images of higher resolution, higher contrast, and lower bias than FBP. This probably also explains the absence of statistical significance between the SRT and Tera-Tomo 3D reconstructions.

Nowadays, 3D-OSEM has become the standard reconstruction method for routine static and dynamic PET imaging employed by most commercial clinical and preclinical systems. Nonetheless, attention must be paid when employing iterative reconstruction methods, since their optimal reconstruction parameters (product of subsets and iterations) for diagnostic PET imaging do not necessarily provide the most accurate quantification [40]. The challenge of assigning and using an IDIF for quantitative analysis of glucose metabolism has resulted in a wide range of published glucose utilization values for the rodent myocardium. It is important to note that in the absence of ground truth data, the Patlak calculations using SRT reconstruction and IVC IDIF compare well with previous calculations using FBP or OSEM reconstructions as well as a variety of IDIFs or AIFs in control mice [14,41].

## 5. Conclusions

In this paper, we presented a quantitative comparison between FBP, the SRT, and Tera-Tomo 3D at various iteration numbers, using preclinical dynamic PET data. The STIR library was employed to reconstruct all the rebinned (2D) sinograms via the FBP and SRT algorithms. Patlak graphical kinetic analysis was employed to evaluate the performance of the algorithm, by calculating myocardial glucose uptake in C57BL/6 mice. Overall, the SRT appears to be comparable to FBP and the variants of the Tera-Tomo 3D algorithm, providing a valid analytic alternative for preclinical dynamic PET imaging applications. In future studies, we intend to combine the SRT algorithm with neural networks (denoiser part) to improve the quality of dynamic PET-reconstructed images, especially the early time frames where the SRT seems to have an advantage.

## Figures and Tables

**Table 1:** Measured metabolic value slope $K_{i}$ (mL/ccm/min) of the applied Patlak kinetic model in the myocardium of seven male C57BL/6 mice (n=7) using FBP, SRT, and the variants of Tera-Tomo 3D algorithm. The VOI analysis was conducted via PMOD software [35].

Slope $K_{i}$: (mL/ccm/min)				
	Reconstruction Algorithm			
Mice	FBP	SRT	Tera-Tomo 4-5	Tera-Tomo 4-13
M1	0.098	0.105	0.091	0.098
M2	0.051	0.054	0.096	0.098
M3	0.055	0.062	0.069	0.069
M4	0.089	0.094	0.090	0.089
M5	0.060	0.065	0.134	0.151
M6	0.077	0.079	0.099	0.102
M7	0.046	0.052	0.101	0.115

**Table 2:** Measured metabolic value MRGlu (μmol/min/100 g) of the applied Patlak kinetic model in the myocardium of seven male C57BL/6 mice (n=7) using FBP, SRT, and the variants of Tera-Tomo 3D algorithm. The VOI analysis was conducted via PMOD software [35].

Metabolic Value MRGlu (μmol/min/100 g)				
	Reconstruction Algorithm			
Mice	FBP	SRT	Tera-Tomo 4-5	Tera-Tomo 4-13
M1	70.50	75.41	64.98	70.49
M2	36.81	38.63	68.84	70.62
M3	39.50	44.14	49.61	49.65
M4	63.82	67.59	64.88	63.66
M5	42.96	46.39	96.34	108.04
M6	55.03	56.92	71.00	73.43
M7	33.10	37.14	72.58	82.68

## Data Availability

The data presented in this study are available upon request from the corresponding author.
